# A Randomized Cross-Over Trial of the Postprandial Effects of Three Different Diets in Patients with Type 2 Diabetes

**DOI:** 10.1371/journal.pone.0079324

**Published:** 2013-11-27

**Authors:** Hanna Fernemark, Christine Jaredsson, Bekim Bunjaku, Ulf Rosenqvist, Fredrik H. Nystrom, Hans Guldbrand

**Affiliations:** 1 Department of Medical and Health Sciences, Faculty of Health Science, Linköping University, Linköping, Sweden; 2 Department of Internal Medicine, Motala Hospital, Motala, Sweden; Universidad Peruana de Ciencias Aplicadas (UPC), Peru

## Abstract

**Background:**

In the clinic setting both fasting levels of glucose and the area under the curve (AUC) of glucose, by determination of HbA1c levels, are used for risk assessments, in type 2 diabetes (NIDDM). However little is known about postprandial levels, and hence AUC, regarding other traditional risk factors such as insulin and blood-lipids and how this is affected by different diets.

**Objective:**

To study postprandial effects of three diets, during a single day, in NIDDM.

**Methods:**

A low-fat diet (45–56 energy-% from carbohydrates), and a low-carbohydrate diet (16–24 energy-% from carbohydrates) was compared with a Mediterranean-style diet (black coffee for breakfast and the same total-caloric intake as the other two diets for lunch with red wine, 32–35 energy−% from carbohydrates) in a randomized cross-over design. Total-caloric intake/test-day at the clinic from food was 1025–1080 kCal in men and 905–984 kCal in women. The test meals were consumed at a diabetes ward under supervision.

**Results:**

Twenty-one participants were recruited and 19 completed the studies. The low-carbohydrate diet induced lower insulin and glucose excursions compared with the low-fat diet (p<0.0005 for both AUC). The insulin-response following the single Mediterranean-style lunch-meal was more pronounced than during the low-fat diet lunch (insulin increase-ratio of the low-fat diet: 4.35±2.2, of Mediterranean-style diet: 8.12±5.2, p = 0.001) while postprandial glucose levels were similar. The increase-ratio of insulin correlated with the elevation of the incretin glucose-dependent insulinotropic-polypeptide following the Mediterranean-style diet lunch (Spearman, r = 0.64, p = 0.003).

**Conclusions:**

The large Mediterranean-style lunch-meal induced similar postprandial glucose-elevations as the low-fat meal despite almost double amount of calories due to a pronounced insulin-increase. This suggests that accumulation of caloric intake from breakfast and lunch to a single large Mediterranean style lunch-meal in NIDDM might be advantageous from a metabolic perspective.

**Trial Registration:**

ClinicalTrials.gov NCT01522157 NCT01522157

## Introduction

Type 2 diabetes is usually a consequence of obesity-induced insulin-resistance which leads to compensatory hyperinsulinaemia and ultimately, when beta-cell failure ensues, to a hyperglycemia of such extent that the diagnosis of diabetes is established. Thus, about 90% of patients develop type 2 diabetes as a consequence of obesity [1] and a majority also display dyslipidemia, hypertension and hyperinsulinemia, which has been denoted the metabolic syndrome, and these patients have an increased risk for premature cardiovascular disease [2]. The American Diabetes Association currently recommends patients with diabetes to consume a diet that has a low content of fat, in particular saturated fats, sugar and salt [1]. However, a few randomized studies in obesity and diabetes have demonstrated that patients compliant with low-fat diets do not increase HDL-cholesterol and reduce fasting serum-triglyceride levels as much as those that are randomized to diets containing less carbohydrates [3]–[6]. Theoretically it might be preferable for patients with type 2 diabetes to avoid carbohydrates since this macronutrient is digested in the gut to form glucose and other sugars, but many experts express concerns for an increased risk of cardiovascular disease consequent to the high intake of fat that carbohydrates are substituted for, and the effects on LDL-cholesterol of high-fat diets have been inconsistent (reviewed in [7]). Another dietary regime that has caught much interest is a food composition based on a traditional Mediterranean eating behavior. Such eating pattern has been associated with low prevalence of cardiovascular disease in epidemiologic investigations, and often the high intakes of vegetables and unsaturated fatty acids in fish and olives have been suggested as crucial components that might reduce cardiovascular disease [8], [9]. Indeed, recently a randomized study, called PREDIMED, showed lower cardiovascular disease when subjects were assigned to a Mediterranean diet, with extra olive oil or nuts, compared to a traditional low-fat diet, and this was also so for the large subgroup of patients with type 2 diabetes [10]. Wine has traditionally been consumed as the beverage to the meals in Mediterranean regions, and a moderate intake of alcohol has been linked with low prevalence of cardiovascular disease and improvement of risk factors [11]–[13], indeed, such advices were also given in the PREDIMED study in the Mediterranean diet groups [10]. However, another aspect that might be of interest for glycemic control is the fact that a traditional meal distribution in countries such as Greece, Portugal and Italy do not provide much calories, sometimes none at all, for breakfast, but rather that a large proportion of the calories is consumed at lunch, or in the evening [14], [15]. This might be a metabolic advantage since the diurnal pattern of the hypothalamic-pituitary-adrenal system renders a peak of plasma cortisol in the morning, and cortisol reduces the sensitivity for insulin. Recently it was shown that concentrating the amount of calories from carbohydrates to dinner-time, rather than to consume this macronutrient at all meals during the day, improved insulin-sensitivity in obese subjects [16].

When glucose and other food components enter the small intestine, entero-endocrine L-cells release incretins such as glucagon-like pepide-1 (GLP-1), while K-cells release glucose-dependent insulinotropic polypeptide (GIP) into the blood [17]. In contrast to GLP-1 [18], the release of GIP is not decreased in patients with type 2 diabetes [19] and it is enhanced not only by glucose, but also by fat and protein [20]. Both these hormones enhance the release of insulin in a glucose-dependent manner.

The very great majority of earlier studies on the effects of different diets on risk factors for cardiovascular disease have focused on fasting levels of blood lipids, glucose and insulin. On the other hand, when treating patients with type 2 diabetes, glycemic targets include levels of HbA1c which corresponds to the area under the curve (AUC) for glucose levels since HbA1c is the amount of glucose that has chemically bound to the hemoglobin molecule [21]. Indeed, HbA1c levels are more often used as markers of good glycemic control than fasting glucose levels [22], [23] since HbA1c levels are closely related to diabetic complications. This is also in line with the increased use of 24-hour blood pressure recordings in the clinic setting of patients with hypertension which has now also been advocated in recent guidelines as an important evaluation tool which strongly predicts cardiovascular risk [24]. However, we are not aware of any trials that have studied effects on postprandial levels, and thus of AUC, on traditional risk factor such as blood lipids and insulin. Assuming that not only the AUC for glucose is of interest for complications of diabetes, but that this might be relevant for such other risk factors, we compared potential differences of a traditional low-fat diet as compared with a low-carbohydrate diet and a Mediterranean-like food (and meal-distribution) in a cross-over design in patients with type 2 diabetes. The aim was thus primarily to compare changes in glucose, insulin and blood-lipids following ingestion of a low- or a high-fat diet with that of a Mediterranean diet in which calories for breakfast in the two comparator diets were ingested as one single large meal for lunch, including a glass of red wine. To gain insights into mechanistic of differences in the diets we also studied effects on the meal-stimulated hormones GIP and the satiation signaling hormone leptin.

## Materials and Methods

The protocol for this trial and supporting CONSORT checklist are available as Supporting Information; see Checklist S1 and Protocol S1.

Suitable patients were recruited by nurses and physicians at the diabetes unit at Motala Hospital and the primary care facilities “Lyckorna” and “Marieberg” which are situated adjacent to the hospital. The patients all had to have type 2 diabetes treated with or without anti-diabetic drugs. However, patients treated with insulin or sulfonylureas could not participate due to the risk of hypoglycaemia when intake of carbohydrates was to be reduced by the diets used for comparison with the low-fat control-diet. The participants received written and oral information about the study and were also asked not to change their regular diets or exercise habits during the complete time-period of the study.

Each patient tested all the three different diets in randomized order. Randomization was performed by drawing ballots and the sequence of the diets was determined at one single occasion for all participants. The maximally allowed time-span between each diet-test was 3 weeks and there had to be at least one day of wash-out between experiments to avoid any carry-over effect. The participants were fasting from 22.00 pm the previous day, when arriving for assessment of each diet. Anthropometric data were collected in the morning and a venous cannula was inserted distally to the cubital fossa on the forearm of the patient from which blood was drawn at 07.30 am (fasting), 09.15 am (corresponding to after breakfast or coffee), 11.00 am (before lunch), 12.45 pm (after lunch), 14.30 pm (late after lunch) and the last sample was taken at 16.15 pm. All study meals were consumed under supervision by the study organizers (HF and CJ) at the Diabetes ward of Motala Hospital.

### Diets

The total caloric intake each of each period of the experimental days at the hospital (not including food eaten at home later during the day) was 1025–1080 kCal in men and 905–984 kCal for the women. During the intervention day the participants were served breakfast, or only black coffee for the day with the Mediterranean food, at 8.00 am and lunch was served at 11.30 am. Food and macronutrient compositions of the three different diets are shown in Table 1. The participants were asked to abstain from exercise or walking during the experiments, and thus they remained in the dining room area during the days of the experiments, in company with the study organizers (HF and CJ). Dinner (evening meal after 16.15 pm) during the trial days was thus eaten according to individual preferences at home.

**Table 1:** Compositions of breakfast and lunch.

	Low-fat diet	Low-carbohydrate	Mediterranen Diet
		diet	
**Breakfast (HE/LE)**	Wholegrain bread	Bacon (40g/30g)	Black coffee (200ml)
	(60g/60g)	2 Eggs	
	Black coffee (200	Swedish ryebread	
	ml)	(24g)	
	Cucumber	Pepper	
	Pepper	Butter (10g)	
		Black coffee (200	
		ml)	

	High-energy	Low-energy	High-energy	Low-energy	High-energy	Low-energy
**Energy (kJ)**	1281 (306 kcal)	1239 (296 kcal)	1834 (438 kcal)	1834 (438 kcal)	17 (4 kcal)	17 (4 kcal)
**Protein (E%)**	18	19	25	25	20	20
**Protein (g)**	13.6	13.8	26.9	26.9	0.2	0.2
**Carbohydrate (E%)**	45	48	16	16	80	80
**Carbohydrate (g)**	33.7	35.3	17.3	17.3	0.8	0.8
**Fat (E%)**	37	33	59	59	0	0
**Fat (g)**	12.9	11.0	29.2	29.2	0.0	0.0
**MUFA (E%)**	12	10	24	24	0	0
**MUFA (g)**	4.2	3.5	11.8	11.8	0.0	0.0
**PUFA (E%)**	5	4	7	7	0	0
**PUFA (g)**	1.8	1.5	3.4	3.4	0.0	0.0
**SFA (E%)**	17	15	22	22	0	0
**SFA (g)**	5.9	5.1	11.0	11.0	0.0	0.0
**Lunch (HE/LE)**	Ricenoodles (100g/80g)		Mixed vegetables (150g/100g)		Tomato (70g/70g)	
	Sweet chili sauce (9g/9g)		Cheese 28% (10g)		Cucumber (40g/40g)	
	Japanese soy (18g)		Swedish ryebread (12g)		Rucola (50g/60g)	
	Chicken (100g/80g)		Cottage cheese (40g/30g)		Olives (38g/38g)	
	Liquid margarine (20g/20)		2 Eggs		White bread (150g/160g)	
	Sugar snap peas (50g/40g)		Butter (15g/10g)		1 piece of garlic (3g/3g)	
	Pepper (60g/30g)		Pear (100/50g)		Salmon (100g/80g)	
	Broccoli (70g/50g)				Red pepper (50g/30g)	
	Red onion (30g)				Cashew nuts (7g/5g)	
	Cashew nuts (10g/5g)					
	1 piece of garlic (3g/3g)				Red wine JP Chenet Cabernet-Syrah	
					14% alc. (200ml/150ml)	

	High-energy	Low-energy	High-energy	Low-energy	High-energy	Low-energy
**Energy (kJ)**	3136 (749 kcal)	2558 (611 kcal)	2458 (587 kcal)	1955 (467 kcal)	4505 (1076 kcal)	4103 (980 kcal)
**Protein (E%)**	17	17	28	28	15	14
**Protein (g)**	31.7	25.0	40.3	32.1	38.8	33.6
**Carbohydrate (E%)**	56	55	24	21	32	35
**Carbohydrate (g)**	103.9	82.6	34.3	24.2	85.6	84.1
**Fat (E%)**	26	29	48	51	40	41
**Fat (g)**	22.4	19.7	32.1	26.9	49.0	45.1
**MUFA (E%)**	16	17	16	17	24	25
**MUFA (g)**	13.2	11.8	10.2	9.0	29.1	27.4
**PUFA (E%)**	5	6	4	4	7	7
**PUFA (g)**	4.5	4.0	2.6	2.3	8.3	7.3
**SFA (E%)**	4	4	23	23	7	7
**SFA (g)**	3.4	2.8	15.5	12.3	8.1	7.3

Women received the low-energy meals (LE) and men received the high-energy (HE) meals. Energy % (E%) was calculated based on protein, fat and carbohydrate content.

Abbreviations: alc, alcohol; E%, energy percent; MUFA, mono-unsaturated fatty acid; PUFA, poly-unsaturated fatty acid; SFA, saturated fatty acid.

### Ethics

This study was conducted according to the guidelines laid down in the Declaration of Helsinki and all procedures involving human subjects/patients were approved by the Regional Ethics Committee of Linköping, Sweden (Dnr 2011/418-31). All participants gave written informed consent to participate. The study was registered with trial number NCT01522157 at ClinicalTrials.gov.

### Statistics

Statistical calculations were done with PASW 18.0 software (SPSS Inc. Chicago, IL, USA). Comparisons between groups were done with pairwise comparisons using Wilcoxon's signed rank paired test with the low-fat diet serving as control in the analyses. The pairwise comparisons of different time points during the test days were only analyzed if there were differences in the AUC as compared with the low-fat diet, except for GIP that is a hormone specifically affected acutely by the meals and which thus was expected to be low before the meals. Spearman's rank was used for calculation of correlations. Mean (SD) is given unless otherwise stated. Statistical significance for paired comparisons was considered to be present as p<0.0063 after Bonferroni correction, i.e. p = 1-(1-α)^1/k^ where α is the traditional p-value of 0.05 and k = 8 (i.e. the number of variables tested). In correlation analyses statistical significance was set as p<0.05. The AUC were calculated based on the trapezoidal rule. The sample size was determined based on the experiences of our earlier long-term study of a low-carbohydrate compared with a low-fat diet in which we found substantial differences between these two groups in a total of 32 subjects that reported reasonable compliance with the diets at two years [4]. In that study we clinically observed larger difference in postprandial glucose levels than in fasting levels of glucose and that it was possible to substantially reduce insulin doses in those compliant with the diets, thus indirectly demonstrating lower insulin requirement by the different diets. Since all participants in the present study should eat the meals of the diets in company with the study organizers in the trial reported here, compliance was assumed to be excellent and hence to give high statistical power, but due to risk of attrition, we planned to include about 20 subjects. We also chose a cross-over design to increase statistical power. As stated in the introduction, we found no published earlier studies in patients with type 2 diabetes of postprandial effects of different diets on which to base a more exact power calculation.

## Results

**Figure 1:**
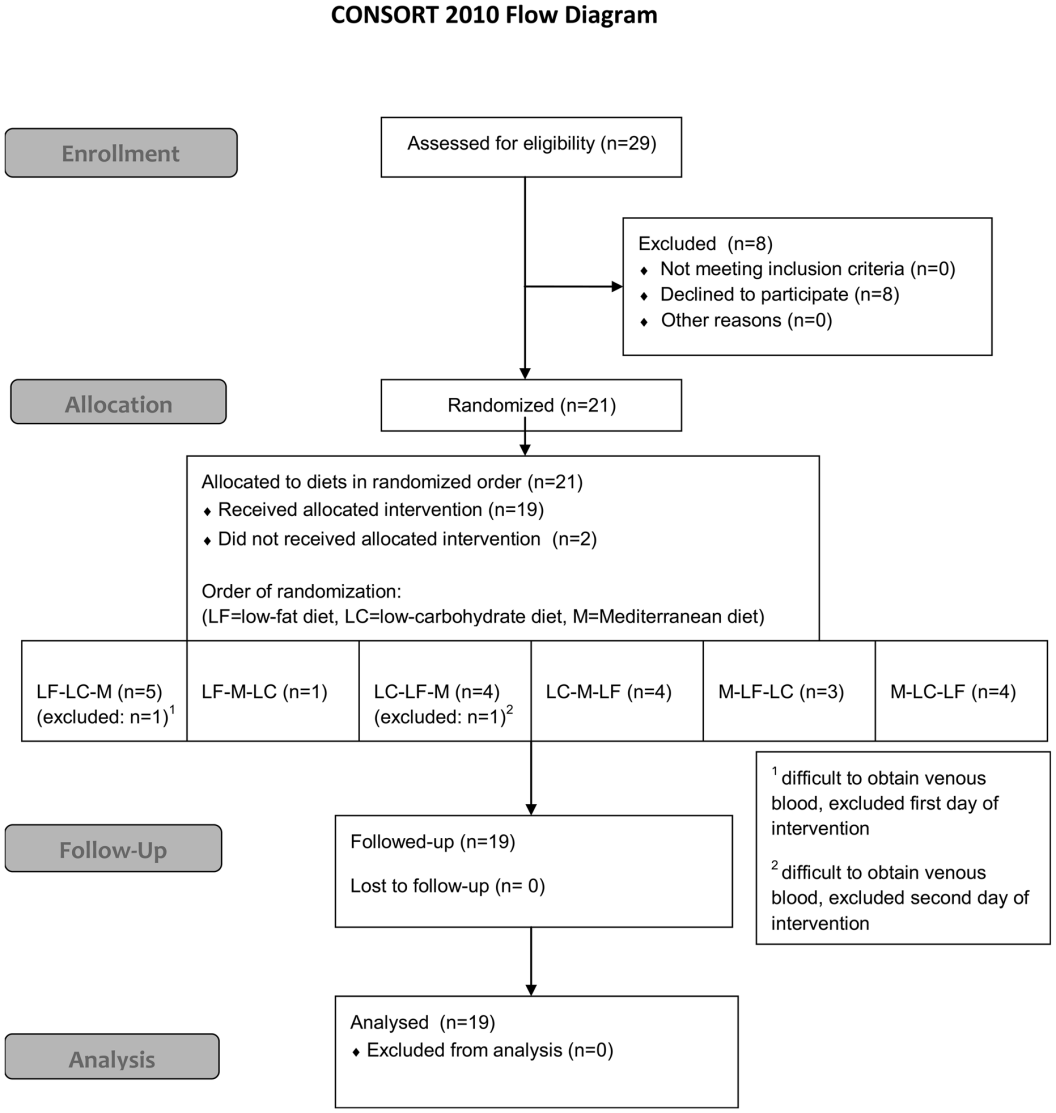
CONSORT Flow Diagram. Abbreviations: LF, low-fat diet; LC, low-carbohydrate diet; M, Mediterranean diet.

Twenty-one patients with diabetes were recruited for the study initially, by local advertising and by direct contact from the study organizers with potentially suitable patients. The study was conducted from February 6 2012 to April 2 2012. In two of the participants it was very difficult to obtain repeated venous blood samples during the first or the second experimental day, and further participation was judged to be impossible. Hence, these participants were excluded from further participation. The remaining 19 participants completed all three experimental days and the baseline characteristics of these patients are shown in Table 2. Figure 1 shows a flowchart of the study. The elapsed time period between each meal varied from 44 hours to 31 days with a mean value of 12±7 days.

**Table 2:** Baseline characteristics of the patients in the morning of the first individual test meal.

Variable (n = 19)		SD
Men/Woman (n/n)	10/9	
Age (year, m)	63	10
Diabetes-duration (year, m)	7	7
Height (cm, m)	168	9
Weight (kg, m)	84.4	17
BMI (kg/m^2^, m)	29.8	4.2
Waist (cm, m)	97	13
Sagittal abdominal diameter (cm, m)	30	5
Systolic BP (mmHg, m)	140	14
Diastolic BP (mmHg, m)	80	12
HbA1c (mmol/mol, m)	51	10
Metformin (n)	8	8
Lipid-lowering treatment (n)	9	9
No medical treatment (n)	3	3

Abbreviations: BMI, body mass index; BP, blood pressure; n, number; m, mean value; SD, standard deviation.

The carry-over effects of the 8 main variables was tested by calculating differences of fasting values and no differences comparing changes between the first and the second test days, nor for the second and the third test days were found (all p>0.08 by Mann-Whitney).

**Figure 2:**
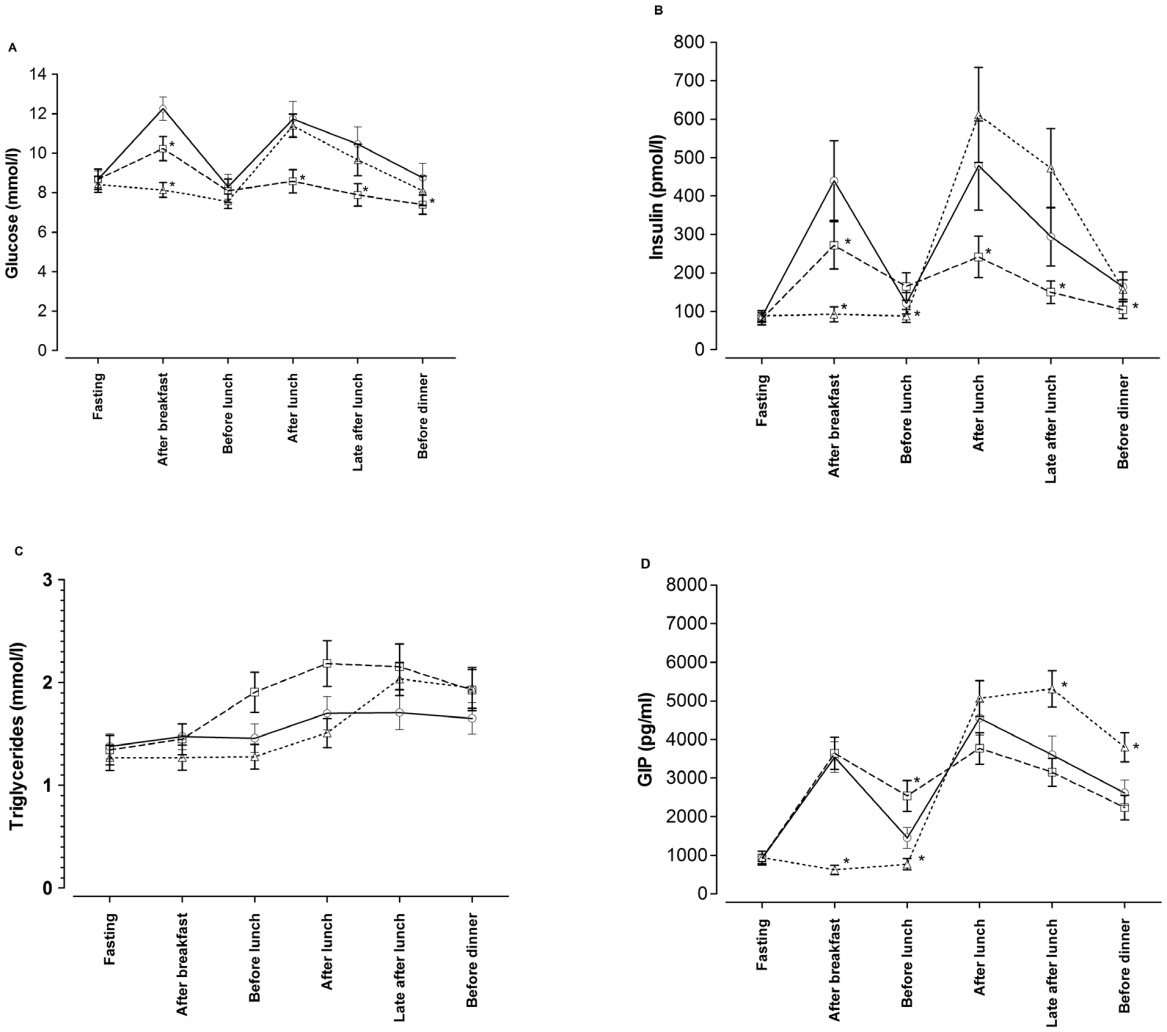
Effects of three different diets on glucose, insulin, triglycerides and glucose-dependent insulinotropic-polypeptide (GIP). Effects of a low-fat diet (open circles, regular lines), low-carbohydrate diet (open squares, dashed lines) and a Mediterranean-type diet (open triangles, dotted lines) with similar total energy intake as the low-fat and low-carbohydrate diets contained for both breakfast and lunch combined but eaten only at lunch paired with a glass of red wine (a, effects on glucose; b, effects on insulin; c, effects on serum triglyceride levels; d, effects on the incretin hormone glucose-dependent insulinotropic-polypeptide). All 19 patients with type 2 diabetes tested the three different diets for breakfast and lunch, *corresponds to Wilcoxon's signed rank paired test in comparison with the control (low-fat diet) at the same time-point (p<0.0063) for those variables that displayed differences in AUC (except GIP, see statistics section).

As seen in Figures 2a and 2b glucose and insulin levels increased more markedly following the low-fat meals compared with the low-carbohydrate meals. Table 3 shows the AUC for insulin, glucose, lipids and the other hormones. The corresponding figures show trends and statistical significances of the specific time-points compared with the low-fat diet as control (total cholesterol, LDL cholesterol, HDL cholesterol and leptin as Supporting Information, Figures S1–S4). The difference in AUC for triglycerides during the low-carbohydrate test day tended to be statistically significantly higher than during the low-fat test day, but the p-value (p = 0.014) did not meet the Bonferroni corrected level for statistical significance (Figure 2c and Table 3).

**Table 3:** Levels of the areas under the curves for the tested variables, standard deviation and the p-value when comparing with the low-fat (control) group at the same time period (Wilcoxon Signed Ranks Test).

Variable	Diet	Area under curve		
			SD	P
Glucose	Low-fat	90	26	
(mmol/l)	Low-carb	75	21	<0.0005
	Mediterranean	80	17	0.001
Insulin	Low-fat	2555	2328	
(pmol/l)	Low-carb	1609	1396	<0.0005
	Mediterranean	2425	1766	0.841
Triglycerides	Low-fat	14	5	
(mmol/l)	Low-carb	16	7	0.014
	Mediterranean	13	5	0.276
Cholesterol	Low-fat	39	9	
(mmol/l)	Low-carb	39	8	0.121
	Mediterranean	39	9	0.705
HDL-cholesterol	Low-fat	12	4	
(mmol/l)	Low-carb	13	4	0.055
	Mediterranean	12	4	0.897
LDL-cholesterol	Low-fat	20	8	
(mmol/l)	Low-carb	20	7	0.199
	Mediterranean	21	8	0.670
GIP	Low-fat	26319	13081	
(pg/ml)	Low-carb	25680	11752	0.879
	Mediterranean	24727	8885	0.215
Leptin	Low-fat	16758	11611	
(pg/ml)	Low-carb	15874	12684	0.557
	Mediterranean	13822	11187	0.001

A p-value of <0.0063 was considered statistically significant.

Abbreviations, Low-carb., low-carbohydrate diet; GIP, glucose-dependent insulinotropic polypeptide; SD, standard deviation.

The levels of glucose and insulin were unchanged by drinking the black coffee in the morning during the Mediterranean-diet, as compared with fasting levels (Figures 2a and 2b). Postprandial glucose levels did not differ when comparing the lunch of the Mediterranean-diet and the low-fat diet (Figure 2a), while the relative increase in insulin (the increase as a ratio, i.e. insulin levels after the meal divided by insulin levels ahead of meal) was higher (insulin ratio of low-fat diet: 4.35±2.2, of Mediterranean diet: 8.12±5.2, p = 0.001)

The incretin GIP increased after the meals and peaked after the large lunch during the Mediterranean-diet after which the levels did not decline as fast as after lunch of the other two diets (Figure 2d). There was a positive correlation between the increase in insulin levels and the corresponding elevation of the GIP levels after the Mediterranean-diet (r = 0.64, p = 0.003, n = 19, corresponding figure for all meals, r = 0.76, p<0.0001, n = 57). The AUC for leptin was lower during the Mediterranean-diet compared with the low-fat diet (Table 3).

## Discussion

We found a low-fat diet to induce higher AUC levels of both insulin and glucose levels as compared with low-carbohydrate diet with a similar caloric content after the two test meals. This is in line with a requirement for large insulin amounts to handle the large carbohydrate intake of the low-fat diet and suggests the low-carbohydrate diet to be preferable regarding glycemic control in patients with type 2 diabetes when compared with a low-fat diet. These findings corroborate our earlier published data of the long-term effects of diets with similar macronutrient compositions as the low-fat diet and the low-carbohydrate diet tested in the study presented herein, since we found glycemic control to improve and insulin requirement to be reduced on a low-carbohydrate diet at 6 months, when compliance was reasonably good with the diets [4]. However, concern has been raised regarding a large intake of fat when treated with a low-carbohydrate diet since this might raise postprandial triglycerides, and there was a statistically non-significant trend (p = 0.014) towards such a finding in our study when comparing the AUC for triglycerides with that of the low-fat diet. Such a finding would be in line with physiological effects that dietary fatty acids are transported in chylomicrons postprandially and also that patients with type 2 diabetes have reduced clearance of chylomicrons, that presumably acts to enhance such effects [25]. Indeed, postprandial levels of triglycerides have been shown to correlate positively with intima-media thickness in type 2 diabetes [26], suggestive of increased risk for cardiovascular disease in this respect. However, it should also be noted that fasting levels of triglycerides have in some studies been lowered more effectively by low-carbohydrate diets compared with low-fat diets [27], [28].

Since it has been common not to consume any calories for breakfast in many Mediterranean regions [14], [15], [29], and to accumulate calories to a large lunch or meal in the evening, we compared such a diet and eating pattern to the low-fat and low-carbohydrate diets in our study. While black coffee for breakfast had no discernible effects of the risk factors measured, the Mediterranean-style diet induced a substantial increase in insulin after the lunch which apparently was large enough to keep glucose excursions to be similar as during the low-fat diet containing much less calories at this single meal. This could be of clinic interest, since a typical feature of type 2 diabetes is a reduced ability to secrete insulin in response to a meal [30]. The increase in insulin after lunch was positively correlated with the corresponding increase in GIP levels, suggestive of that at least a part of release of insulin during the Mediterranean-diet at lunch could be due an incretin-effect to enhance the glucose-stimulated release of insulin. However, such a correlation does not prove causality, and also other effects of incretins, such as delayed emptying of the ventricle, might have affected glucose levels. Furthermore, the Mediterranean-diet included a glass of wine, and alcohol is known to cause vasodilatation and to reduce glucose production, which could have affected hormonal and glucose responses. The pronounced increase in GIP following the lunch of the Mediterranean-diet could be a consequence of the large amounts of nutrients reaching the small intestine *per se* since not only glucose but also protein and fat induce release of GIP in humans [20]. Indeed, the ingestion of few large meals rather than several smaller ones to achieve a more efficient GIP-release has earlier been advocated by other authors [19].

Leptin levels decreased specifically following the lunch in the Mediterranean-diet and the AUC of leptin was lower on the day of the low-carbohydrate diet compared with the low-fat diet. This could potentially lead to increased appetite but also to less induction of metabolic rate, since leptin has effects to reduce sense of hunger and to increase metabolism. However, resistance to these normal actions of leptin have been demonstrated in obesity [31]. Leptin has also been shown to harbor sympathomimetic effects and the serum levels correlate independently with left ventricular mass [32], [33], and thus reduced levels of this hormone could speculatively be advantageous from a cardiovascular perspective in this sense. The decreased leptin levels following the large amount of nutrients on the Mediterranean-lunch are corroborated by earlier finding that longer cooking-time to enhance nutrient availability reduced leptin levels postprandially in type 2 diabetes [34]. The data are in contrast, however, with findings of increased levels of leptin by red wine [35].

We acknowledge several shortcomings of our trial. We did not aim to recruit patients in a randomized manner to accomplish a group of patients that were representative of most patients with type 2 diabetes in our region. The reason for this was mainly that we prioritized knowledge of the selected patients to be compliant with all the three study days. Indeed, of the recruited 21 patients, only two did not complete the study, and this was due to technical reasons. The intake if the meals under supervision at the diabetes ward rendered excellent compliance which of course reduces the generalizibility of the tested meals if these were instead to be used as diet suggestions in the clinic. Another shortcoming was that patients returned to regular eating-habits in between the three trial days, and that we had no control of diets eaten on the days ahead of trial days. However, our main objective was to compare the response of the different diets on an individual level by the cross-over design, and under regular living conditions we also assume that quite large changes in macronutrient composition do occur since dietary regimes can vary from day to day. The main aim of our trial was to study postprandial effects, calculated as AUC, on traditional risk factors for cardiovascular disease. The determinations of levels of leptin and GIP were added to explore potential mechanistics since these hormones are acutely affected by food intake. Since there were no earlier studies on these hormones of similar design as ours, we had no way to determine statistical power of the observations, consequently we suggest that the results from the analyses of these hormones should be viewed upon as a pilot study.

From a biological perspective we assumed that there would not be any carry-over effect since at least 36 hours had to be passed between lunch on a test day and the corresponding occasion in the fasting state that started a subsequent test day, with regular eating pattern as wash-out in between. This was also confirmed in the statistical analyses of potential carry-over effects. The shortest wash-out period in any participant was 44 hours.

In summary we found that breakfast and lunch based on a low-fat diet compared with a low-carbohydrate diet showed larger postprandial glucose and insulin increases resulting in larger AUC levels of these variables. However, the low-carbohydrate diet showed a trend towards higher levels of triglycerides, which was expected physiologically. To further delineate the clinical implication of these findings, long-term randomized trials with hard-endpoint would be required to compare the effects of the differences between diets. Finally we did not find evidence that accumulation of caloric intake from both breakfast and lunch to one single large lunch, consumed together with extra calories from red wine, enhanced glucose excursions compared with that of a low-fat diet, but rather that insulin-release was improved after this single meal. This suggests that further studies of the diurnal distribution of caloric intake could be of interest in type 2 diabetes.

## Supporting Information

Figure S1 **Effects of three different diets on total cholesterol,** HDL-cholesterol, LDL-cholesterol and the satiating hormone leptin. Effects of a low-fat diet (open circles, regular lines), low-carbohydrate diet (open squares, dashed lines) and a Mediterranean-type diet (open triangles, dotted lines) with similar total energy intake as the low-fat and low-carbohydrate diets contained for both breakfast and lunch combined but eaten only at lunch paired with a glass of red wine (effects on total cholesterol). All 19 patients with type 2 diabetes tested the three different diets for breakfast and lunch in a randomized cross-over design, *corresponds to Wilcoxon's signed rank paired test in comparison with the control (low-fat diet) at the same time-point (p<0.0063) for those variables that displayed differences in AUC as seen in Table 2.
(TIF)

Figure S2 **Effects on LDL-cholesterol.** All other information same as Figure S1.
(TIF)

Figure S3 **Effects on HDL-cholesterol.** All other information same as Figure S1.
(TIF)

Figure S4 **Effects on leptin.** All other information same as Figure S1.
(TIF)

Protocol S1 **Application for ethical vetting of research involving humans.** The application approved by the Regional Ethical Review Board in Linköping, Sweden.
(DOCX)

Checklist S1 **CONSORT 2010 checklist.** Information of included data in the randomized trial.
(DOC)
