# Supplementary material for: A Randomized Cross-Over Trial of the Postprandial Effects of Three Different Diets in Patients with Type 2 Diabetes
Source: PLoS One. 2013 Nov 27;8(11):e79324. doi: 10.1371/journal.pone.0079324 (PMC3842308; doi:10.1371/journal.pone.0079324)
Supplement: Protocol S1 — Application for ethical vetting of research involving humans. The application approved by the Regional Ethical Review Board in Linköping, Sweden. (DOCX) [file pone.0079324.s005.docx]

**APPLICATION FOR ETHICAL VETTING OF RESEARCH INVOLVING HUMANS** 1

**APPLICATION FOR ETHICAL VETTING OF RESEARCH INVOLVING HUMANS**

**For information concerning the application, see *Guide to the application (www.epn.se)***

IT IS NOT PERMITTED TO USE THE ENGLISH APPLICATION FORM OR TO FILL IN THE APPLICATION IN ENGLISH. THE SWEDISH APPLICATION FORM MUST BE USED.

**Depending upon what research the application concerns, the information asked for below will have various degrees of relevance. In cases of changes to a previously approved application, see *Guide to the application*.**

**To the Regional Ethical Review Board in:** *Linköping, Sweden*

State the Regional Ethical Review Board to which the entity principally responsible for the research belongs. For a list of boards see *www.epn.se*.

Date fee paid:

Please note that an application is never complete (and thus able to be processed) until the form is correctly filled in and the fee has been paid.

**Project title:** *Acute effects of different diets on blood lipids and blood glucose*

Give a descriptive title in Swedish for laymen, without using confidential information. Where suitable, for instance in cases of clinical trials of medicinal products, also state the identity of the project and the number, date, version etc. of the project/research plan (protocol or testing plan). In the case of changes to a previously approved application, see Guide to the application.

Project number/identity: Version number:

EudraCT nr (for clinical trials of medicinal products):

***Information to be completed by the Regional Ethical Review Board***

Application complete: Case number:

Request for additional information concerning the application: Requested information received:

Date of decision: Date processed:

**The application concerns (also applies when an advisory statement is requested): APPLICATION FOR ETHICAL VETTING OF RESEARCH INVOLVING HUMANS** 2

research in which only one responsible research body participates (5 000 kr)

research in which more than one responsible research body participates (16 000 kr)

research in which more than one responsible research body participates,

but in which all the researchers or subjects of research have an immediate

link to only one of the responsible research bodies (5 000 kr)

only processing personal data (5 000 kr)

(When only existing population registers, e.g. national databases, will be used)

research involving clinical trials of medicinal products (16 000 kr)

changes to a previously approved application in accordance with p. 4 of Statute

(2003:615) concerning the Ethical Review of Research Involving Humans (2 000 kr)

If the board decides that the legislation concerning ethical review is not applicable to the study/research project, is an advisory statement wanted? (See p. 4a and 4b in Statute 2003:615 and Guide to the application)

Yes: No:

**1. Information concerning the body principally responsible for the research etc.**

**1:1 The responsible research body** (See p. 1:1 in Guide to the application)

The application for ethical vetting of research is to be made by the responsible research body. *By responsible research body is meant a government authority or a physical or legal entity under whose auspices the research is conducted.*

Name: *Department of Endocrinology Linköping University Hospital.*

Address*: 581 85 Linköping*

**1:2 Qualified representative of the body principally responsible for the research**

A qualified representative can be, for example, head of department, head of unit or head of operations. The responsible research body is to decide itself by internal consultation, delegation, or by means of power of attorney, who is qualified to act as a representative of the body.

Name: Official title: *Henrik Hjortswang*

Address: *Department of Endocrinology Linköping University Hospital., 581 85 Linköping*

**1:3 The researcher/s primarily responsible for the conduct of the project**

**(principal contact person/s)** (See p. 9, annex 10 and p. 1:3 in Guide to the application)

Note! It is the principal researcher who is responsible for ensuring that others carrying out the project have sufficient competence (scientific as well as clinical) and, in the case of clinical trials of medicinal products, are **APPLICATION FOR ETHICAL VETTING OF RESEARCH INVOLVING HUMANS** familiar with “Good Clinical Practice” (GCP). When work is being carried out by doctoral candidates, the principal supervisor is the principal researcher who bears the main responsibility for the conduct of a project, as a rule.

Name: *Fredrik H Nyström Official title: Professor, consultant*

Address: *Department of Endocrinology Linköping University Hospital 581 85 Linköping*

E-mail: *fredrik.nystrom@lio.se*

Telephone: *0101037749*

Mobile phone: *0736 569303*

**1:4 Other participants** (See p. 9, annex 1 and p. 1:4 in Guide to the application)

Other responsible research bodies, plus researchers responsible for completing the project locally (principal contacts) are to be listed here or in an annex stating names and addresses (see p. 9, annex 1).

*Motala Hospital, Sweden.*

**1:5 Describe access to necessary resources during the course of the project**

(See p. 9, annex 9 and p. 1:5 in Guide to the application)

List the person/s (head of department, director of department etc.) who will be responsible for the research participants’ safety at all units/clinics where they will participate. Testimonials from this/these responsible party/ies must be appended (see p. 9, annex 9). The testimonial/s should state that the required economic, structural and personnel resources are available to guarantee the safety of the research participants.

*Hans Guldbrand, GP primary health care centre Lyckorna in Motala*

**1:6 Applications/notifications to other authorities as applicable**

(See p. 1:6 in Guide to the application) **Submitted Date**

a) Clinical testing of medicinal products: Swedish Medical Products Agency _________ *NA*

b) Handling of personal data concerning genetic dispositions: Swedish Data Inspection Board _________*NA*

c) Establishment of a biobank: Swedish National Board of Health and Welfare _________ *NA*

d) Research involving ionising radiation: :radiation protection committee(s) _________ *NA*

**2. Information concerning the project**

**2:1 Summary of the research project** (See p. 9, annex 2 and p. 2:1 in Guide to the application) **APPLICATION FOR ETHICAL VETTING OF RESEARCH INVOLVING HUMANS** 4

The description must be comprehensible to all board members. It is therefore advisable to avoid specialist terminology. State the background and the purpose of the study, together with the scientific question(s) for which answers are being sought. State the most important variables for the investigation. State what advances in knowledge can be expected as a result of the project and what the significance of these might be. State if it is a study of records, if the research is commissioned, etc. Detailed information in the research plan, protocol or programme that is intended for specialists must be appended as an annex (see p. 9, annex 2). For information on what to include in the research plan/protocol, p. 2:1 in Guidance to the application. Include when the data collection is expected to be complete. A more detailed description that is *intended for laymen* concerning the implementation of the study can, if needed, be appended to the obligatory research plan intended for experts.

*It is clinically fully established that high blood lipids and high blood glucose are risk factors for heart and vascular disease that can be successfully treated with various drugs. Blood lipids are analyzed by tradition in the fasting state, because the possibility to capture the subjects own production of various fats (lipids). It has been noted that during a large part of the day subjects are in a fed state recently eaten a larger or smaller amount of calories. Based on the assumption that blood lipids by themselves in the state of dyslipidemia can cause atherosclerosis, it seems reasonable that also high concentrations of lipids after such a meal could cause atherosclerosis in the long term. This is something that has been studied to a very little extent. If compared with blood glucose as part of the clinical assessment of risks, not only the fasting blood glucose but also the blood glucose levels after a meal is important in the control of diabetes. In the assessment and treatment of high blood pressure so-called 24-hour blood pressure recordings are used in order to take into account the total pressure load during the day when assessing risk.*

*Traditionally, since several decades, many agencies (national food administration) have advocated a low-fat and hence high-carbohydrate diet. Meanwhile, many modern studies that compared this diet with a diet with more fat in proportion to the energy from carbohydrates often proved more effective to reduce blood glucose levels in diabetes, and to have beneficial effects on the levels of HDL cholesterol. All these effects have been studied preferably with fasting samples and thus the knowledge is small about the more acute effects of low-carbohydrate or carbohydrate-rich diet on lipids after eating. There is a discussion today about the diet that may be considered appropriate from cardiac and vascular standpoint, and many studies suggest that a so-called Mediterranean diet is preferable. The Mediterranean diet is not a well-defined diet and the traditional Mediterranean diet, for example ate in Crete in the 70s, given special attention to the extremely low incidence of heart disease, was linked to so large amounts of olive oil that it is difficult to replicate today (1 liter / week for middle-aged men) and also comprised a relatively large portion of the calories of alcohol as wine (Retsina). Alcoholic beverages are included in a traditional Mediterranean diet, and in the current SBU report "Food in Diabetes" in 2010 highlighted a moderate alcohol intake as a protective factor in diabetes. We have recently published that a moderate alcohol intake lowers LDL cholesterol by about 10% (Kechagias et al. Annals of Medicine 2011). A third factor in the true traditional Mediterranean diet, which is not so often studied, is that eating breakfast hardly existed at all. The day often started with a cup of coffee without any food at all.*

*We now want to carry out a study of the acute effects of three different diets for breakfast and for lunch on blood lipids and blood glucose. Our research question is whether blood glucose and lipids (risk markers for cardiovascular disease) after meals influenced by different proportions of carbohydrate and fat even though it contains the same amount of energy. We have some experience with this from previous studies in healthy volunteers and then noticed that it is quite small changes in the number of people who are young and fit. To increase the clinical relevance of our study, we therefore focus on patients with type 2 diabetes who are not treated with insulin. We would thus include about 20 participants to study the effects of a traditional diet of food, where approximately 55-60% of calories come from carbohydrates, and where the same proportion of calories provided in the morning and for lunch. For comparison, we plan to let the energy from fat to be 50% in a low carbohydrate diet with a maximum of 20% of energy from carbohydrates. Finally, participants on a third occasion should eat a really traditional Mediterranean diet with no other breakfast than a cup of coffee and then the same amount of calories as in the other two occasions in total for lunch (about 1200 kCal) where meal drink consisting of wine. Blood samples for quantitative determination of the level of glucose and fats are taken before and after these meals so participants will be fasting when coming to our study facility where we prepared the food. Participants will need to stay a few hours after lunch meal so they will be on our study facility from morning to afternoon. Help with adequate repatriation will be arranged on the day drinking wine at lunch. For the participants, the study will provide information on blood glucose and lipid reactions. This information will be given individually by the study at a meeting with one of the physicians who ran the project.*

**2:2 What is/are the primary scientific question(s) forming the basis of the design of the**

**project?**

If the project can be described as testing a hypothesis, state the primary hypothesis and the secondary hypothesis if there is one. Referral can be made to more detailed information for experts in the form of an appended research plan in accordance with 2:1.

*Are risk markers for cardiovascular disease affected in a different way when the same amount of energy in the diet is given in different proportions of carbohydrate and fat?*

**2:3 State the results from relevant animal experiments (clinical trials)**

If animal experiments have not been carried out, the reasons for this must be given.

*Not applicable.*

**2:4 Give an overview of the examination procedures used, data collection and the nature of**

**the data** (See p. 9, annex 5 and p. 2:4 in Guide to the application)

It should be clear from the description how it is planned to complete the project. Describe the nature of data collected. Describe how the reliability of the data is ensured (e.g. quality control/monitoring). When questionnaires and interviews are used, the procedure used should be described and, for example, the content of questions and how conclusions are drawn. Questionnaires and rating scales must be appended (see p. 9 annex 5). For medical research it should be stated, for example, the types of intervention, the methods of measurement, the number of visits, the time required each time research is carried out and the means used to administer any medicinal products and/or isotopes and the amount of blood samples taken (including the accumulated amount when multiple testing takes place). State also if the examination procedure etc. differs from routine clinical measures, and if so, in what way. If a treatment is being studied for the first time on humans, this should be stated and the relevant safety measures described. State which procedure can be required to administer the prospective treatment after the conclusion of the study. State the procedures used to collect biological material. Account for data sources and procedures when processing personal data. For more detailed information, referral can be made to the appended research plan. **APPLICATION FOR ETHICAL VETTING OF RESEARCH INVOLVING HUMANS** 5

*The study includes survey data (VAS scale) describing the participants experience of the different diets and blood samples for the study of risk markers and inflammation. Blood samples will be taken on 6 occasions every test day (see study protocol) with total blood volume <100 ml. We also will collect data on body weight, blood pressure, abdominal circumference, and clinical data, including medications.*

**2:5 Describe how collected biological material is to be stored in a biobank**

(See p. 2:5 in Guide to the application)

*By biobank is meant biological material that has been collected and retained from one or more persons, the origins of which can be traced to the person or persons from which it originates and is being kept pending further notice or for a certain period of time.* Describe where and how each sample to be retained will be kept, the coding procedures and what conditions apply when material is released. State who the person responsible for the biobank is.

*Blood for later analysis of lipids and hormones stored in -80C freezer situated at pharmacological department, U.S. Fredrik Nystrom is responsible for the blood samples for analysis. Data will be processed anonymously. Responsible for biological material is the same as listed above.*

**2:6 Report the necessary resources during project implementation**

Specify who is responsible (director or equivalent) for research subjects' safety at all units / clinics where research subjects will participate. Certificate from those responsible must be attached (see p. 9 Appendix 9). The certificate must state that the requisite economic, structural and human resources are available to ensure the safety of research subjects.

*Staff resources at the University and the County Council's project, see the certificate.*

**2:7 Documentation, data protection and record-keeping** (See p. 2:7 in Guide to the application)

State how the examination procedures and any operations that take place are to be recorded. State if audio and video recordings are to be used. If the material is to be coded, state the procedure: who will retain the code list /code-key in safe-keepingand which person or persons have access to them; where they are being kept and for how long; and if the material will be rendered anonymous or destroyed. Describe how accessible the data material is, how it is kept and how the requisite confidentiality is attained.

*Code list of linking anonymous data with actual data / personal number performed by PI Fredrik Nyström. No medical work and no medical procedures are performed, and thus there is no medical record keeping in the study. The data will be saved at the University Hospital. Depersonalised data files will be handled within the University (HU). If there are samples that arises suspicion of disease this will be handled in the usual clinical manner at the clinic with usual record keeping and investigation outside the study where Fredrik Nystrom has the responsibility.*

**2:8 Describe previous experience (your own and/or others’) of the procedure, technique or**

**treatment used**

It is particularly important that the risks of complications are clearly accounted for and that the relevant publications are listed. When patients are to be given a new form of treatment, such as a pharmaceutical product, it should be stated how many patients (with the complaint in question or another one), had previously been given the proposed treatment, dosage of a pharmaceutical product (or other dosage) and over which treatment periods the treatment has been studied.

*Fredrik Nyström (and Hans Guldbrand) have conducted studies in healthy volunteers and in patients with type 2 diabetes where similar studies were carried out, but then mainly in fasting conditions. Patients are not exposed to risks other than their ordinary living other than that which is made up of relatively large blood sampling. Risks associated with this in patients already before used to giving blood samples should be minimal.*

**3. Information about the research participants**

**3:1 How are research participants chosen?** (See p. 9, annex 3 and p. 3:1 in Guide to the application)

*By research participant is meant a living person who is the subject of the research.* State the selection criteria (inclusion and exclusion). Give an account of the manner in which the researcher will get in contact with/become aware of suitable participants for the research. If advertising is to take place, the advertising material must be submitted as an annex (see p. 9 annex 3). If, for example, children or people who, temporarily or permanently, are not capable of themselves giving their informed consent are to participate in the project, this is to be specifically justified. If certain groups are to be excluded from the project, this is to be specifically justified. **APPLICATION FOR ETHICAL VETTING OF RESEARCH INVOLVING HUMANS** 6

*Inclusion criteria: Patients with type 2 diabetes not treated with insulin or sulfonylureas, which are relatively well controlled due to blood glucose (HbA1c below 75 mmol / mol).*

*Exclusion criteria: Physical or mental problems that make participation difficult to implement. Poorly controlled diabetes with the risk of substantial changes in blood glucose.*

**3:2 State the relationship between the researchers/leader of the research and those participating in the research**

Person administering the treatment (e.g. doctor, psychologist, physiotherapist) - person participating in the research (e.g. patient, client)

Course facilitator (teacher) - student

Employer - employee

Any other relationship that could possibly entail a risk of influence. Describe:

**3:3 State the statistical foundation with respect to the size of the population(s) and/or**

**material(s) studied** (See p. 3:3 in Guide to the application)

Give an account of the statistical power, the so-called power-calculation, or give an account of equivalent considerations which clarify the study's ability to answer the questions posed.

*Study size is based on the few previous studies in this area where effects are found in healthy volunteers of the planned investigations. Patients with diabetes may exhibit greater variability in blood glucose, but we also assume that the distribution of the reaction may be increased as well as various forms of medication will be affected. Therefore, we estimate that we need to study 20 people, which is a number that corresponds to what have been used in previous related studies.*

**3:4 State if participants in the research may be included in several studies, either**

**simultaneously or in another study or other studies closely linked to this one. If so, what**

**kind of research?** (See p. 3:4 in Guide to the application)

*No it is not intended.*

**3:5 What insurance cover is there for research participants taking part in the project?**

It is the responsibility of the entity principally responsible for the research to check that existing insurance policies cover any injuries that may arise in connection with the research.

*We can not see that the study means any real risks, but hospital insurance covers complications of venous sampling.*

**3:6 What financial remuneration or other benefits are participants in the research entitled**

**to and when is this to be paid? (A more detailed description can be submitted as an annex.)** (See p. 9, annex 11 and p. 3:6 in Guide to the application)

Compensation for discomfort and inconvenience - amount (before tax):

Compensation for income from employment Yes No

Allowance for travelling expenses Yes No

Exemption from costs of pharmaceutical products Yes No

Exemption from other costs - which?

Other benefits - which? Compensation for sampling: *1500 SEK in total.*

When is compensation paid? *On completion of the study.*

No compensation to be paid **APPLICATION FOR ETHICAL VETTING OF RESEARCH INVOLVING HUMANS** 7

**4. Information and consent**

**4:1 The procedure involved and the content of the *information* that is given when subjects**

**are asked to participate in the research** (See p. 9, annex 4 and Information for research participants)

In accordance with section 16 of the Act (2003:460) concerning the Ethical Review of Research Involving Humans, research participants are to be informed of the overall plan for and purpose of the research, the methods that will be used, the consequences and risks the research can entail, who the entity principally responsible for the research is, that participation is voluntary, and that they can end their participation at any time. Describe how and when the information is given and what it contains. Indicate who provides the information. Brief and easily comprehensible written information should normally be given. This written information must be attached to the application (see p. 9 annex 4). If no information or incomplete information is given, detailed reasons for this must be given.

*The subjects will be informed orally and in writing of the study by representative who meets them for recruitment purposes.*

**4:2 How is *consent* to be obtained and from whom?** (See Information for research participants)

Describe the procedure: who asks, when this takes place and how the consent is documented. Exhaustive

documentation is particularly important, not only when children or people with a diminished ability to make

decisions participate in the study but also when the study involves a group or groups such as school classes, associations, organisations, companies, church communions, congregations, or groups that interact within social media.

*Documentation by written informed consent, see Appendix.*

**5. Considerations in the light of research ethics**

**5:1 Describe the risks that participation might entail**

This could mean, for example, physical injury, pain, discomfort or other violations of personal integrity that the

project entails or can entail, both long-term and short-term. State not only which measures been taken to prevent the risks mentioned above, but also what preparations have been made to deal with these complications. Give an account of the methods that will be used to investigate, register and report undesirable events.

*Participants are exposed to minimal risks. As we only will recruit patients who are not treated with insulin or other medicines that can cause low blood glucose, we will cause almost no increased risk at all compared to their usual living other than in the form of increased blood sampling.*

**5:2 Describe the predictable benefit for the people participating in the research who are**

**part of the project (applies especially to treatment)**

*Experience in participating in a research project, knowledge of the levels of various cardiovascular risk factors / blood tests and how they are affected by different kinds of diets.*

**5:3 In a broader perspective, identify and specify which ethical problems, such as risk**

**versus benefit, can arise as a part of or as a result of the project** (See p. 5:3 in Guide to the application)

*We believe that the scientific value of the study and interest in the individual participant is greater than the very small risk exposure in patients.*

**APPLICATION FOR ETHICAL VETTING OF RESEARCH INVOLVING HUMANS** 8

Here, for example, an account can be given of which groups (besides the research participants within the research project) can be identified or helped as a result of the study.

*In the longer term differences in the diets more acute effects can affect the dietary advice given to patients with type 2 diabetes.*

**6. Presenting the results**

**6:1 How are both the entity principally responsible for the research and research**

**collaborators guaranteed access to data (to be stated when the research is commissioned) and who is responsible for processing data and writing reports?** (See p. 6:1 in Guide to the application)

*Fredrik Nystrom is responsible for data collection and for reporting in scientific papers in peer-reviewed international journal.*

**6:2 How will the results be made publicly available? Will the study be sent for publishing in**

**a journal or published in some other manner?** (See p. 6:2 in Guide to the application**)**

State the format in which it is planned to make the results public and indicate the time frame that is applicable.

*Scientific reports in peer-reviewed international journals.*

**6:3 In what manner will the right to integrity of those participating in the research be**

**guaranteed when the material is made public or is published?**

Will results be shown at a statistical group level? Describe the procedures or the methods used for concealing identities or ensuring anonymity.

*Results are presented for groups. All presentations are completely anonymous.*

**7. Reporting the financial circumstances and dependencies**

**The purpose of the accounts to be given in points 7:1-7:3 is to clarify all direct or indirect circumstances that could possibly affect the researcher's relationship to the persons participating in the research (during the process of information, consent or implementation, for example).**

**7:1 When the research is commissioned**

State who the commissioning body is: for example a company (when there is clinical testing of pharmaceuticals or the testing of other new products, for example) an organisation or an authority.

Name: Contact for the commissioning body: Not applicable.

Address: Telephone/mobile phone: **APPLICATION FOR ETHICAL VETTING OF RESEARCH INVOLVING HUMANS** 9

State the relationship between the responsible research body for the research/participating researchers and the commissioning body the research (employee/employer, for example).

**7:2 Give an account of any financial agreements with a responsible body or any other financiers**

**(name, amount)**

Where the clinical testing of pharmaceuticals is concerned, reference should be made to agreements entered into with the responsible health-care body. Similar agreements may occur when other research is commissioned and should be accounted for in the same way. Separate agreements with the person or persons who are to carry out the research are also to be presented. State the amount that is allocated for the study/compensation to the clinic/person carrying out the research and what the compensation is to cover. Any sums given to persons participating in the research should also be accounted for here.

*Not applicable.*

**7:3 Give an account of the interests of the responsible research body, the principal researcher and of participating researchers**

An account is to be given here of, for example, ownership of shares, employment status, consultancy work for companies providing finance and any companies owned by the researchers that could benefit financially, directly or indirectly, as a result of the research.

There are no known such vested interests in this study.

**8. Signatures**

Authorized representative for the responsible research body which is making the application, according to 1.2

Place: Date:

Signature: __________________________________________________________________

Clarification of signature:

Official title:

The undersigned researcher who is carrying out the project (principal researcher) according to.1:3 hereby certifies that the research will be carried out in accordance with the application.

Place: Date:

Signature: _________________________________________________________________ **APPLICATION FOR ETHICAL VETTING OF RESEARCH INVOLVING HUMANS** 10

Clarification of signature:

Official title: **APPLICATION FOR ETHICAL VETTING OF RESEARCH INVOLVING HUMANS** 11
